# Numerical Study of a Novel Kagome-Inspired Photonic Crystal Fiber-Based Surface Plasmon Resonance Biosensor for Detection of Blood Components and Analytical Targets

**DOI:** 10.3390/bios15080539

**Published:** 2025-08-15

**Authors:** Ayushman Ramola, Amit Kumar Shakya, Ali Droby, Arik Bergman

**Affiliations:** 1 Department of Electrical and Electronics Engineering, Ariel University, Ariel 40700, Israel; ayushmanr@ariel.ac.il (A.R.); arikb@ariel.ac.il (A.B.); 2 Physics Program, Graduate Center of the City University of New York, New York, NY 10016, USA; adroby@gradcenter.cuny.edu

**Keywords:** surface plasmon resonance, photonic crystal fiber, kagome lattice, hollow core, blood biomarkers, analytical targets

## Abstract

This numerical study introduces a surface plasmon resonance (SPR)-based biosensor utilizing a kagome lattice-inspired hollow core photonic crystal fiber (PCF) for the highly sensitive detection of various blood biomarkers and analytical components. The sensor is designed to detect key blood biomarkers such as water, glucose, plasma, and hemoglobin (Hb), as well as analytical targets including krypton, sylgard, ethanol, polyacrylamide (PA), and bovine serum albumin (BSA), by monitoring shifts in the resonance wavelength (RW). A dual-polarization approach is employed by analyzing both transverse magnetic (TM) and transverse electric (TE) modes. The proposed sensor demonstrates exceptional performance, achieving maximum wavelength sensitivities (*Sw*) of 18,900 nm RIU^−1^ for TM pol. and 16,800 nm RIU^−1^ for TE pol. Corresponding peak amplitude sensitivities (*S_A_*) of 71,224 RIU^−1^ for TM pol. and 58,112 RIU^−1^ for TE pol. were also observed. The peak sensor resolution (*S_R_*) for both modes is on the order of 10^−6^ RIU, underscoring its high precision. Owing to its enhanced sensitivity, compact design, and robust dual-polarization capability, the proposed biosensor holds strong promise for point-of-care diagnostics and real-time blood component analysis.

## 1. Introduction

The successful detection of blood biomarkers using photonic crystal fiber (PCF)-based surface plasmon resonance (SPR) biosensors represents a significant advancement in the medical diagnostics space, enabling a more nuanced understanding of the health of any given individual. Blood comprises several key components, including white blood cells (WBCs), red blood cells (RBCs), plasma, platelets, and hemoglobin (Hb) [1,2,3,4]. Each component plays a crucial role in maintaining bodily functions. The detection and analysis of these components are essential for diagnosing and monitoring a wide range of medical conditions. Their detection with PCF-SPR biosensors has the potential to revolutionize healthcare by enabling the early detection of diseases, personalized medicine, and continuous health monitoring.

Besides blood biomarkers, several analytical targets like krypton, sylgard (PDMS), ethanol, polyacrylamide (PA), and bovine serum albumin (BSA) are detected from the proposed sensor model [5,6]. These targets analytes are useful in biosensors for blood testing and related applications. Krypton is considered a rare gas, likely used to simulate low refractive index (RI) analytes for sensing capability [7]. Sylgard is a polymer which is used in microfluidic biosensors to simulate sample handling environments [8]. Ethanol is considered as a popular common solvent which is used to test RI response within the biological blood components [9]. PA is used in the biochemical experiments and serve as a synthetic biomolecule mimic [10]. BSA is a standard model protein in biosensor research to test biomolecule detection, commonly used to simulate protein biomarkers present in the blood [11].

SPR enables the precision measurement of RI changes associated with different blood components and target analytes, which may allow for the identification and timely treatment of specific health issues [12,13,14,15,16,17,18]. Therefore, a PCF-based SPR biosensor for the monitoring of various blood components in an effort to facilitate this early detection of disease in humans is presented in this research [19,20]. Blood components like RBCs transport oxygen to body tissues and transfer CO_2_ back to the heart, and a lack of these cells is the defining feature of anemia [21,22]. WBCs can combat infections, and leukopenia is defined by their presence at lower levels [23]. Similarly, platelets play a critical role in blood clotting and wound healing, and their overly low levels result in thrombocytopenia [24]. Plasma carries proteins, water, enzymes, salts, and other compounds throughout the body, and abnormal plasma homeostasis can cause hypoalbuminemia [25]. Monitoring these blood components can thus help preserve healthy bodily functions.

**Figure 1:**
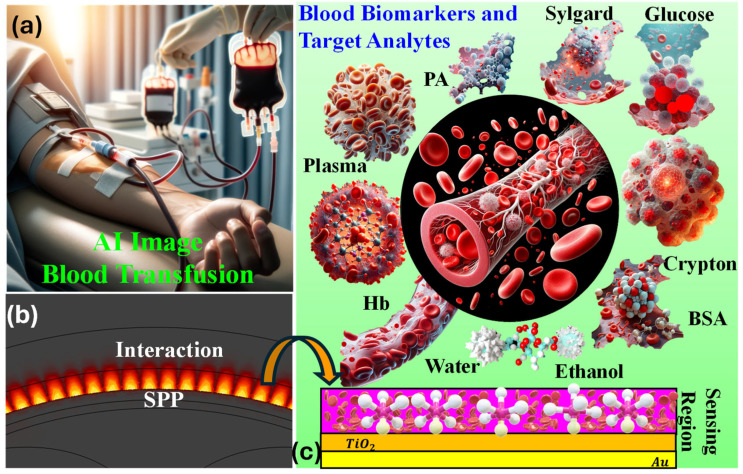
(**a**) AI-based image of blood transfusion in human body. (**b**) Interaction between the blood component plasma with plasmonic material for SPP generation. (**c**) Artistic view of the various blood components and target analytes.

PCF-SPR biosensors excel in detecting subtle variations in RI, a capability essential for distinguishing among different factors in the blood. Their application spans medical disciplines, aiding in the identification of a range of health markers such as genetic abnormalities, glucose levels, cancer detection, cholesterol conditions, and diseases, including COVID-19 and diabetes [26,27,28]. Figure 1a presents an AI-based image of blood transfusion, Figure 1b presents the interaction of blood components with plasmonic material, and Figure 1c presents the classification of various blood components and target analytes.

Recently, various SPR biosensors have been developed for the detection of specific blood components. Chaudhary et al. [29], for instance, designed a PCF-SPR biosensor with a coating of titanium dioxide (TiO_2_) and gold (Au) to distinguish between RBCs, WBCs, Hb, plasma, and water in blood samples. Similarly, Rajeswari et al. [30] developed a PCF-SPR biosensor utilizing indium tin oxide (ITO) for the detection of blood plasma by adjusting variations in water and plasma concentrations within blood samples. Jabin et al. [31] also presented a design for a PCF-SPR biosensor for Hb detection.

Current research in this field aims to discover novel plasmonic substances, expand mode configurations, and boost sensitivity levels for prepared sensors [32]. The design architectures of PCF-SPR biosensors traditionally fall into one of three primary models: external metal deposition (EMD) [33], internal metal deposition (IMD) [34], and the D-shaped framework [35]. Classical plasmonic materials such as Au, copper (Cu), aluminum (Al), silver (Ag), and graphene have been favored in prior studies [36,37]. However, more recent efforts have explored the use of cutting-edge materials, including MXenes [38], transition metal dichalcogenides (TMSCs) [39], transparent conductive oxides (TCOs) [40], magnesium fluoride (MgF_2_) [41], perovskites [42], silicene [43], and phosphorene [44], as well as metal alloys like Au-Ag, Au-Cu, and Au-TiO_2_ [45,46]. Evaluating novel combinations of materials is thus an important area of work for PCF-SPR biosensor design. These efforts involve analyzing various sensing mechanisms using the fundamental principles of coupled-mode theory to model light propagation in both transverse magnetic (TM) and transverse electric (TE) modes. The design and analysis of PCF-SPR sensors can be carried out using the finite element method (FEM), which enables a detailed examination of mode behavior, sensitivity, geometric optimization, and material analyte interactions. Additionally, it facilitates the exploration of birefringence, dispersion, loss metrics, overall durability, and efficacy of the sensor. Measurement protocols such as wavelength and amplitude interrogations can further be applied to discern resonance wavelength (RW) variations between various blood components, thereby equipping PCF-SPR biosensors with the ability to rapidly detect and monitor blood components and associated target analytes. Accordingly, in this article, an EMD-based kagome inspired PCF-SPR sensor model is presented along with exhaustive analysis of the sensor parameters for the effective detection of various blood components and associated target analytes.

## 2. Geometric Configuration of the Biosensing System

**Figure 2:**
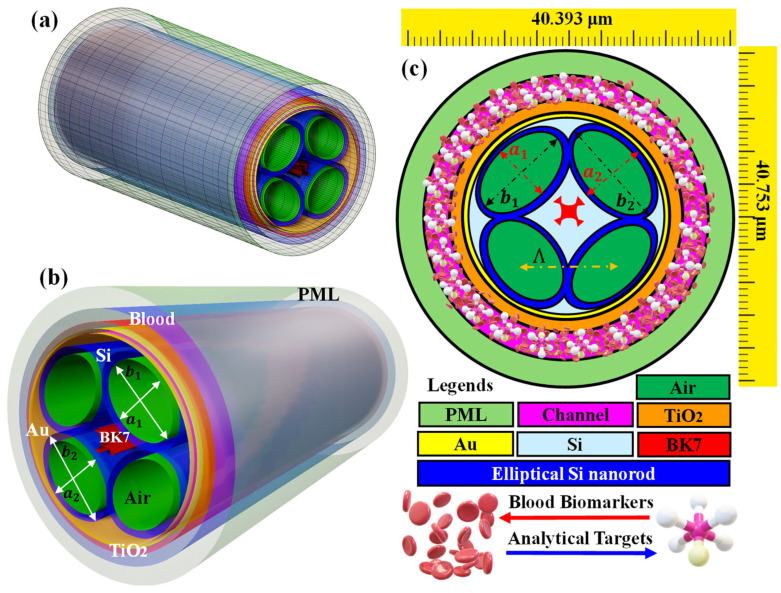
Sensor geometry (**a**) 3D model, (**b**) 3D model of the fiber with design variations, (**c**) classification of the kagome-lattice-inspired SPR sensor.

Figure 2 shows the proposed kagome-inspired hollow-core PCF-SPR biosensor that is designed for the detection of various blood components and target analytes. The hollow-core PCF consists of silica (SiO_2_) glass surrounded by air holes and other features in the cladding. The main advantages of the hollow-core PCF are listed as [47].

***(i) Enhanced light–metal interactions***—Hollow-core PCF models allow light to be guided through an air-filled core. This enhances the evanescent field’s interaction with the surrounding plasmonic material. This may also strengthen the sensor’s sensitivity due to the changes in the RI of the analyte [48].

***(ii) Improved sensitivity***—The hollow-core configuration allows light to interact directly with the plasmonic materials and analyte, which increases the sensitivity to even small RI changes. This is particularly useful for applications like biosensing or chemical detection, where trace quantities of substances need to be detected [48].

***(iii) Reduced dispersion***—Hollow core PCFs often exhibit low dispersion, which helps to maintain stable SPR conditions over a broader wavelength range, potentially enabling multi-wavelength sensing [48].

Hollow core PCFs were chosen for maximum sensitivity and strong analyte interactions. The operational wavelength of the proposed biosensor is within the infrared range (780 nm–2400 nm). The NIR operational range can vary depending on the polarization of the incident light (i.e., TE pol., TM pol.), as polarization affects plasmonic excitation and shifts in RW. Moreover, PCFs with asymmetric cores or coated channels also support SPR for both polarizations at different resonance peaks. Fused SiO_2_ and SCHOTT-BK7 are selected to improve electron excitation and coupling incident light for surface plasmon wave (SPW) generation. This is because a strong interaction between the core and surface plasmon polaritons (SPPs) is required for SPR generation [49,50,51,52].

The geometrical design of the proposed sensor consists of four elliptical air holes with semi-minor axes a_1_ = 13.75 µm, a_2_ = 13.90 µm semi-major axes b_1_ = 18.75 µm, b_2_ = 18.95 µm (Tables S1–S4). The internal and external ellipses create a hollow nanorod, which is used as an air channel in this biosensor. The distance between the centers of two ellipses is the pitch (Λ) = 19.75 µm.

This arrangement of air holes was selected as it will increase the lossiness of the core-guided modes. SiO_2_ is used as the base material in the sensor model. The BK7 glass is used to form a kagome lattice in the middle of this fused SiO_2_. BK7 glass is further selected as the base material for forming the kagome lattice structure. It possesses excellent optical, mechanical, and fabrication properties. Optically, BK7 exhibits a relatively low RI and high transparency across the visible and NIR spectrum, minimizing background absorption and ensuring efficient light guidance within the PCF [53]. The RI contrast between BK7 and the plasmonic layer (Au/TiO_2_) enhances phase matching between the guided core mode and the SPP mode, thereby improving coupling efficiency and sensitivity. Additionally, compared to silica, BK7 has a lower thermal expansion coefficient and a higher melting point, making it more dimensionally stable under thermal variations [54]. It is also stronger and lighter than silica glass, which aids in mechanical robustness and facilitates the fabrication of complex kagome lattice geometries. Due to these combined optical, thermal, and mechanical advantages it is widely used in a large fraction of optical products ensuring high sensor performance, stability, and reproducibility in practical applications.

The other plasmonic materials in this biosensor consist of a combination of Au and TiO_2_. Au is one of the most desirable plasmonic materials due to its high stability and chemically inert nature. It possesses strong SPR activity, enabling high sensitivity to RI changes. Its biocompatibility makes it ideal for biosensing. Furthermore, Au surfaces can be easily functionalized for specific analyte detection, enhancing sensor selectivity. The optimized thickness of Au for this sensor is 45 nm. A thin layer of TiO_2_ is placed over the Au layer as it offers unique advantages due to its high RI enhancing phase-matching for improved sensitivity. It also provides desirable photocatalytic properties, which keep the sensor surface clean and functional over extended periods. TiO_2_ is biocompatible and chemically stable for biosensing applications, ensuring durability. The optimized thickness of the TiO_2_ layer is determined to be 85 nm. A third sensing layer with a thickness of 1.50 µm is used to analyze blood components and target analytes. This layer is the analyte sensing layer of the proposed biosensor. Finally, a perfect-matched layer (PML) of 1.80 µm is installed to reduce light reflections at the sensor boundaries.

The optimized geometrical dimensions of the sensor are determined through a series of calculations that evaluate various sensor configurations and examine the relationship between sensing performance and structural thickness. At these optimized dimensions, the sensor achieves maximum sensitivity while remaining suitable for practical fabrication.

Figure 2a shows the three-dimensional (3D) view of the proposed kagome design-driven PCF-SPR biosensor. The arrangement over the kagome lattice consists of two elliptical holes. The overall design of the sensor model is further repeated beneath the kagome lattice as presented in Figure 2b. The sensor’s geometrical dimensions are selected through rigorous simulation to achieve optimal performance. Figure 2c presents the two-dimensional (2D) cross-sectional view of the biosensor fiber, with classification of the various design parameters.

The RI of SiO_2_ and BK7 are expressed using the Sellmeier equation (Equation (1)) [27,28,55].

(1) $$n^{2}\left(\lambda \right)=1+\sum_{\;1\leq j\leq 3}\frac{B_{j}\lambda^{2}}{\lambda^{2}-C_{j}}$$

**Figure 3:**
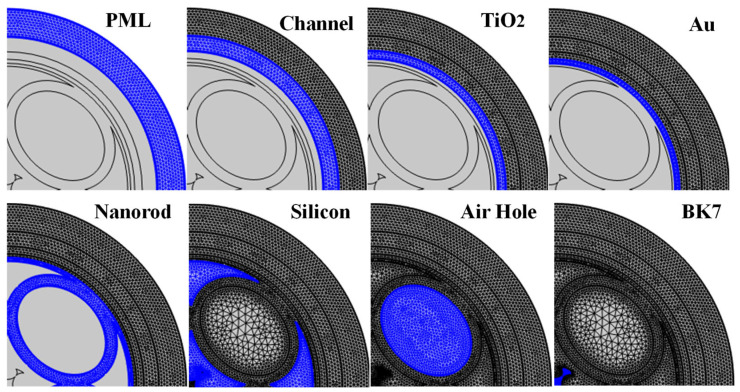
Mesh configuration for the proposed biosensor.

*n*(*λ*) is the RI at wavelength *λ* while *B_j_* and *C_j_* represent the Sellmeier coefficients, which for BK7 are *B*_1_ = 1.03961212, *B*_2_ = 0.231792344, *B*_3_ = 1.01046945, *C*_1_ = 6.00069867 × 10^−3^ µm^2^, *C*_2_ = 2.00179144 × 10^−2^ µm^2^, and *C*_3_ = 103.560653 µm^2^, while for fused SiO_2_, the corresponding values have been published previously [56]. The RI of the Au is obtained with the Drude Lorentz model (Equation (2)) [55].

(2) $$\epsilon \left(\omega \right)=\epsilon_{\infty }-\frac{\omega_{p}^{2}}{\omega \left(\omega +i\gamma_{D}\right)}+\sum_{j}\frac{S_{j}\omega_{0j}^{2}}{\omega_{0j}^{2}-\omega^{2}-i\omega \gamma_{j}}$$

where *ϵ*(*ω*) is frequency-based permittivity, *ϵ*_∞_ is the permittivity at infinite frequency, *ω* is the angular frequency of the incident light, *ω_p_^2^* represents plasma frequency, *γ_D_* is the free electron damping coefficient, and *S_j_*, *ω_0j_^2^*, and *γ_j_* represent damping constants for *j^th^* Lorentz oscillator. The values of these standard constants for Au have been published previously [52,55], as the RI of TiO_2_ mentioned in [57]. The RI of air is 1.000293 RIU, as obtained from the Ciddor equation [58]. Sensor computation is performed through FEM with the COMSOL software package [59]. User-controlled free triangular meshing conditions are used to mesh the components, as represented in Figure 3. Air holes, Au, and TiO_2_ components are meshed using an extremely fine mesh. BK7, fused SiO_2_, sensing layer, and PML are meshed through extra fine mesh conditions. In total, 81,645 triangular elements, 8478 edges, and 389 vertices are meshed. The domain element minimal grid integrity is 0.4184, the element aspect ratio is 0.001963, and the total area of the meshed region is 1646.13 µm^2^. Different mesh conditions provided an optimal balance between computational efficiency and accuracy. The sensor structure is simulated using the scattering boundary condition (SBC) [59].

### 2.1. Production Feasibility Assessment for the Proposed Biosensor

Numerous methods exist for crafting biosensor models, with prominent ones being the sol–gel method [60], injection molding [61], and the stack and draw method [62]. The stack-and-draw method is favored for its cost-effectiveness and adaptability. This method involves aligning capillaries of specific dimensions into a pre-determined layout by stacking them together. They are then heated and merged, followed by drawing them into fibers within a fiber drawing tower. Subsequently, the fibers are cooled and encased in a protective layer.

For the deposition of metals like Au and TiO_2_, alternative methods such as thermal evaporation, end-face polishing, sputtering, electroless plating, and chemical vapor deposition (CVD) are utilized [55]. Among these, CVD is particularly valued for its straightforwardness in the context of sensor model production. There are several additional advantages to using elliptical air holes rather than the conventional circular air holes in the sensor model, listed as follows.

***(i) Polarization control***—Elliptical air holes allow for more precise control over the polarization of the guided modes. This can improve the sensor’s response in both TM pol. and TE pol., which ultimately enhances sensitivity [63].

***(ii) Mode confinement***—Elliptical holes can provide stronger confinement of the electric field near the metal–dielectric interface layer. This leads to better interaction with plasmonic materials like Au and TiO_2_, which is essential for effective SPR biosensing [63].

***(iii) Tailored dispersion properties***—Elliptical air holes offer better flexibility in controlling the dispersion and birefringence of the PCF. This ability improves the sensor’s wavelength selectivity and enhances its response to detect specific blood components [63].

Real-time fabrication of the proposed PCF with elliptical air holes can be performed by implementing the following steps consecutively.

#### 2.1.1. Initial Preparation

Thin-wall capillaries: Initially, thin-wall capillaries that will later form the structure’s air holes are constructed.

Solid rods and stacking: The thin-wall capillaries are stacked with solid rods to create the initial preform mold for the PCF. For this sensor, elliptical capillaries can be created by slightly flattening round capillaries or adjusting their shapes during stacking.

#### 2.1.2. Structuring Elliptical Air Holes

The elliptical shape of the air holes can be achieved by controlling the temperature and pressure during the drawing process, or by pre-structuring elliptical capillaries before stacking. Precise control over shape is crucial to achieve the desired dimensions and orientation of the ellipses.

#### 2.1.3. Stacking

Carefully stacking the elliptical capillaries and solid rods in the desired arrangement is performed to achieve the kagome-lattice pattern.

This stacked layout forms the preform that will be drawn into a fiber. Ensuring proper alignment and maintaining the elliptical shapes are both important at this stage.

#### 2.1.4. Drawing Process

The stacked preform is heated to a high temperature in a furnace, which softens the glass tubes.

The structure is drawn into a fiber while maintaining the elliptical shape of the air holes. This step requires precise control over the drawing speed and temperature to prevent the elliptical shapes from collapsing or distorting.

#### 2.1.5. Plasmonic Coating (CVD) Process)

**CVD TiO_2_:** A thin layer (optimum thickness) of TiO_2_ is applied using CVD. TiO_2_ enhances the RI contrast and can improve coupling with the metal layer.

**CVD Au:** An Au layer is deposited on the outer walls of the elliptical air holes or the outer surface. The Au layer is essential as it provides the SPR effect necessary for biosensing.

#### 2.1.6. Post-Processing

The PCF is inspected to evaluate the consistency of the elliptical shape, as well as uniformity in the TiO_2_ and Au coatings.

As structural defects or irregularities in the elliptical holes can affect the sensor’s performance, quality control at this stage is crucial.

#### 2.1.7. Final Assembly and Sensing Setup

The fabricated PCF is integrated into the biosensing setup, connecting it to a light source, an optical spectrum analyzer (OSA), and a computer for data acquisition and analysis.

**Figure 4:**
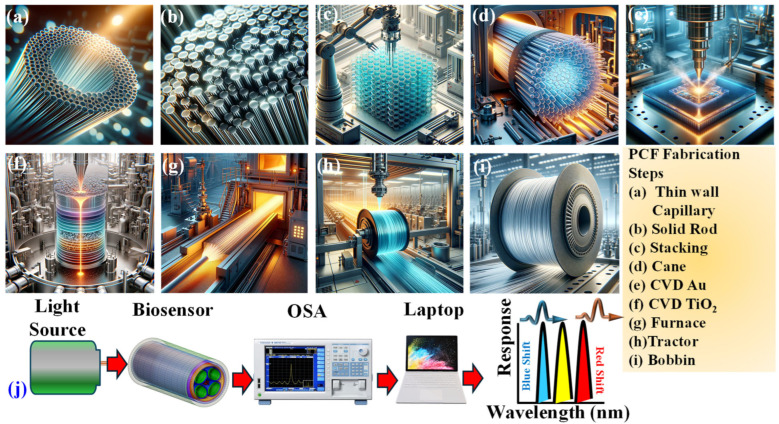
(**a**–**i**) Schematic overview of the PCF fabrication process (**j**) analyte sensing setup.

Figure 4a–i present an overview of the PCF fabrication process, and Figure 4j represents the analyte sensing setup.

Table 1 presents the RI values for different blood components and target analytes analyzed to assess sensor performance in this study

## 3. Methodology and Analysis of Findings

**Figure 5:**
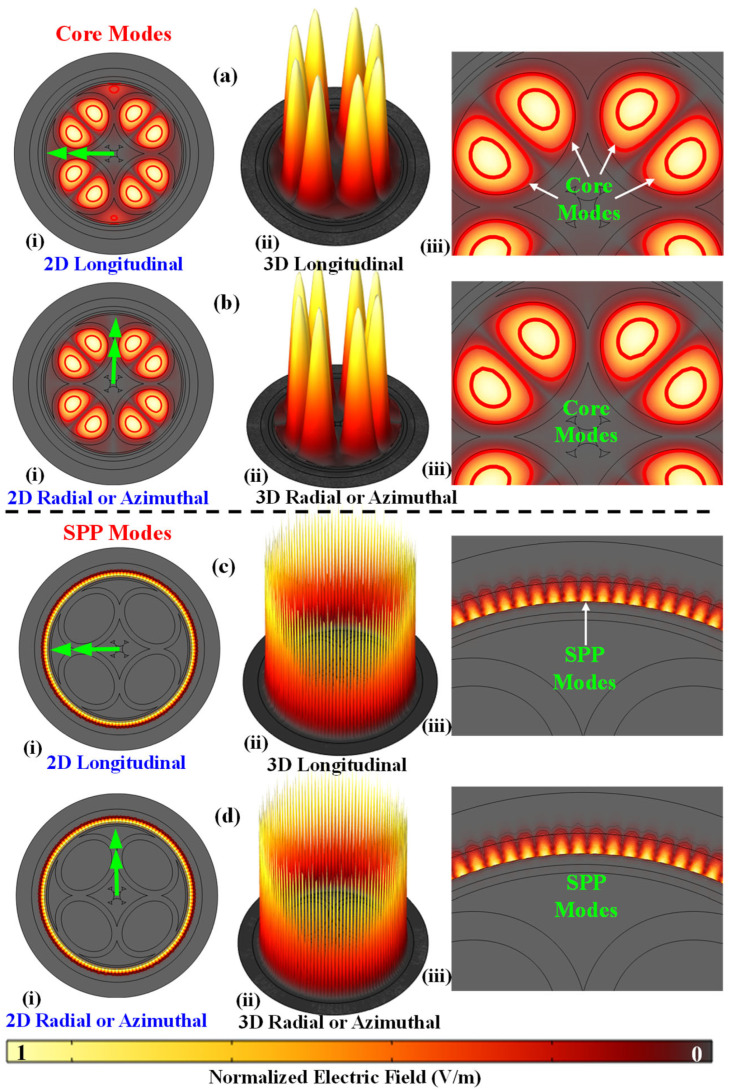
Field distribution intensity profile of the proposed sensor for target analyte BSA TM pol. core mode (**a**) (i) 2D longitudinal (x-pol.), (ii) 3D longitudinal (x-pol.), (iii) zoom view core mode; TE pol. core mode (**b**) (i) 2D radial (y-pol.), (ii) 3D radial (y-pol.), (iii) zoom view core mode; TM pol. SPP mode (**c**) (i) 2D longitudinal (x-pol.), (ii) 3D longitudinal (x-pol.), (iii) zoom view SPP mode; TE pol. SPP mode (**d**) (i) 2D radial (y-pol.), (ii) 3D radial (y-pol.), (iii) zoom view SPP mode.

In the PCF-SPR sensor framework, light predominantly localizes in two critical zones—the core region and the metal–dielectric interface. The incident light is trapped in the hollow core region and assists in the creation of the core mode, while at the interface, it gives rise to the SPP mode. These two modes interact at a key interaction point (i.e., RW). According to the coupled-mode theory, light propagation takes place in optical fibers in two principal directions, commonly referred to as TM pol. (longitudinal) and TE pol. (radial or azimuthal). The interaction between the core mode and the SPP mode may be more pronounced in one of these orientations. However, as the goal of this study is to develop a comprehensive understanding, both TM pol. and TE pol. are investigated in detail. Figure 5a,b present TM pol. and TE pol. field distributions for BSA at RWs of 2382 nm and 2365 nm, respectively. Figure 5c,d show the SPP mode profiles for TM pol. and TE pol., respectively. Similar profiles for other blood components and target analytes can also be obtained. Electric field distribution and mode coupling for this PCF-SPR system can be explained by coupled mode theory as expressed by (Equations (3) and (4)), respectively [57].

(3) $$\frac{dA_{1}}{dz}=i\beta_{1}A_{1}+ikA_{2}$$

(4) $$\frac{dA_{2}}{dz}=i\beta_{2}A_{2}+ikA_{1}$$

where *A*_1_ and *A*_2_ represent the amplitudes of the interacting modes, with *z* denoting the spatial coordinates along the direction of propagation. The propagation constants for these modes are denoted by *β*_1_ and *β*_2_, while the coupling coefficients, *k*, enable their interaction. When the real parts of *β*_1_ and *β*_2_ match, they result in phase-matching, which is crucial for energy transfer between the modes, triggering SPR, which occurs when light waves interact with plasmons at a metal-dielectric interface. The interaction between the SPP and core modes leads to a coupled mode propagation constant (*β*_1_ and *β*_2_) calculated using (Equation (5)) [57].

(5) $$\beta_{\pm }=\beta_{avg}\pm \sqrt[{2}]{\delta^{2}+k^{2}}$$

where *β_±_* represents the propagation constant, *β_avg_* is the average of *β*_1_ and *β*_2_, *k* is the coupling strength, and *δ* is given by *δ* = (*β*_1_ − *β*_2_)/2 in which the imaginary components are denoted by *δ_r_* and *δ_i_*.

There are several reasons that higher-order SPP modes were favored over lower-order modes for this study, and they can be listed as follows.

***(i) Enhanced sensitivity***—Higher-order SPP modes typically exhibit stronger field confinement and greater evanescent field penetration depths compared to lower-order modes. This makes them more sensitive to RI changes near the sensor surface, which is essential for detecting small variations associated with biomolecules in biosensing applications.

***(ii) An improved signal-to-noise ratio (SNR)***—Higher-order modes offer a more pronounced resonance shift in response to analyte binding, leading to better SNRs. This improves the reliability and accuracy of measurements, especially in cases where precise detection of analytes is required, as in the case of blood component analyses.

***(iii) Optimal mode overlap with the analyte region***—In PCF-based SPR biosensors, higher-order SPP modes are more likely to overlap with the analyte region where the target biomolecules are present. This overlap increases the interaction between the evanescent field and the target components, thus enhancing the sensor sensitivity.

***(iv) Better control over polarization and propagation characteristics***—Higher-order modes allow for more control over the polarization (radial, azimuthal, or longitudinal) and propagation characteristics of the electromagnetic fields. This flexibility can be advantageous for optimizing the design based on specific detection requirements or sample characteristics.

In the proposed kagome-lattice PCF-SPR sensor, the choice of higher-order SPP modes is motivated by the specific waveguiding characteristics of the kagome structure. The kagome lattice exhibits a large air-filling fraction and reduced confinement in the cladding, which allows the higher-order guided core modes to extend further toward the plasmonic coating. This increased modal overlap with the metal–dielectric interface enhances the phase-matching condition between the core mode and the SPP mode, leading to stronger evanescent field interaction with the analyte region. Furthermore, the lower confinement loss (*α_CL_*) for certain higher-order modes in the kagome geometry enables longer interaction lengths and sharper resonance dips, which directly translate into higher wavelength and amplitude sensitivity (*S_A_*). Thus, in the context of the kagome-lattice waveguide, the use of higher-order SPP modes is not only beneficial for sensitivity enhancement in general but is intrinsically supported by the structure’s ability to sustain and efficiently couple these modes to the plasmonic surface.

A simulation video (Video S1) is provided as Supplementary Material to illustrate the electromagnetic field distributions of various TM and TE modes supported by the proposed PCF SPR sensor. This video offers a dynamic visualization of both low-order and higher-order TM and TE modes, showcasing the propagation behavior and field confinement characteristics within the sensor structure. The temporal evolution of the fields is animated to show resonance conditions, and confinement dynamics in real time. Furthermore, the video transitions between different mode orders and polarization states to enable comparative analysis under varying external RI conditions. This visualization enriches the understanding of modal interactions with the plasmonic layer offering a more intuitive grasp of the sensor’s performance.

### 3.1. Determination of Biosensor Characteristics

In PCF-SPR biosensors, the core mode *α_CL_* represents the extent of light intensity attenuation occurring as the incident light propagates within the core of the PCF. It quantifies the leakage of light from the core mode into the cladding or surrounding medium due to coupling with lossy plasmonic modes. This loss is especially important for characterizing sensor sensitivity and resonance behavior. This loss significantly influences the sensitivity, detection capabilities, and overall operational efficacy, as represented by (Equation (6)) [55].

(6) $$\alpha_{CL}(dB/cm)=8.686\times (2\pi /\lambda )\times Im(n_{eff})\times 10^{4}$$

where *Im(n_eff_)* represents the imaginary component of the effective RI and *λ* is the wavelength in nm. Mode coupling occurs at the RW, achieving its peak value as detailed in Table 2. Blood components and target analytes can be distinguished by examining their specific TM pol. and TE pol. core mode wavelengths. 

**Figure 6:**
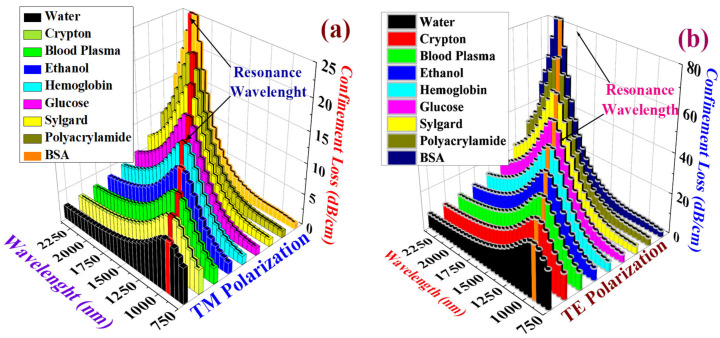
*α_CL_* behaviors of different blood components. (**a**) TM pol. (**b**) TE pol.

Figure 6a,b illustrate the *α_CL_* behavior for both TM pol. and TE pol., demonstrating that blood components and target analytes exhibit unique *α_CL_* peaks at different RWs. Table 2 provides a consolidated overview of the key insights derived from Figure 6a and Figure 6b, which, respectively, correspond to the TM pol. and TE pol. Here, Δ*α_CL_* and |RW| signify the change in the *α_CL_* and RW values between TM pol. and TE pol. This information can be used to derive additional statistical information about the behavior of these modes along different polarizations.

One can conclude from Table 2 that, due to changes in $\alpha_{CL}$ and RW for different blood components and target analytes, a variation in RW with respect to the RI is observed. This variation is quantified using wavelength sensitivity (*Sw*), as defined by (Equation (7)) [55].

(7) $$S_{W}(nm/RIU)=∆\lambda_{P}/∆RI$$

where the RW shifts between two individual blood components and target analytes are measured by ∆RI and $∆\lambda_{P}$, respectively, representing the changes in RI and RW. The ∆RI between various blood components and target analytes can be obtained from Table 1.

Using these approaches, the obtained *Sw* values for TM pol. corresponding to water, crypton, plasma, ethanol, Hb, glucose, sylgard, and PA are 17,100, 17,300, 18,900, 10,100, 10,550, 1333.33, 11,818.18, and 16,388.88 nm RIU^−1^, respectively.

Similarly, the *Sw* values for TE pol. corresponding to water, crypton, plasma, ethanol, Hb, glucose, sylgard, and PA are 14,900, 15,200, 16,800, 8900, 9200, 6600, 9363.63, and 14,888.88 nm RIU^−1^ respectively. The average *Sw* values for TM pol. and TE pol. are 12,936.29 nm RIU^−1^ and 11,981.56 nm RIU^−1^, respectively.

**Figure 7:**
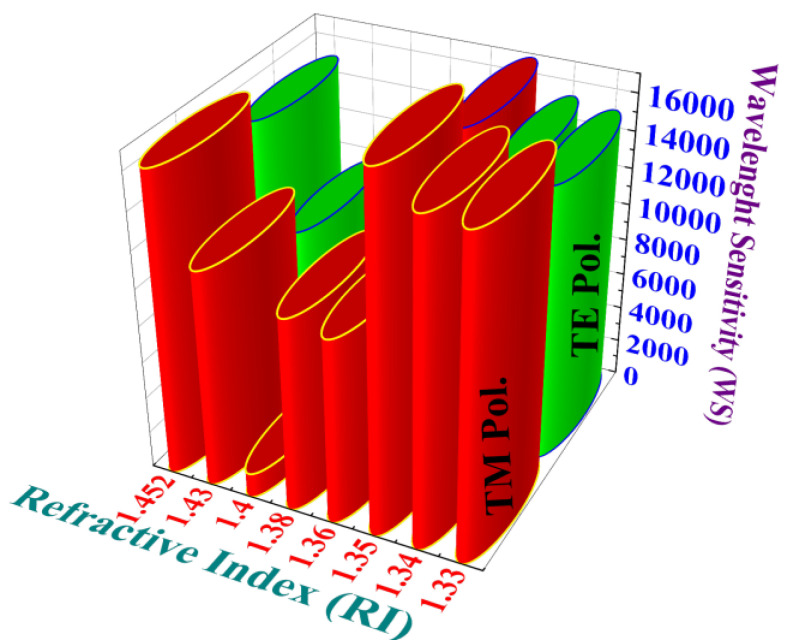
Comparison of *Sw* values for TM pol. and TE pol.

Figure 7 shows the comparisons of *Sw* values for TM pol. and TE pol., respectively. These results revealed high *Sw* values in both cases, although the maximal values were evident for TM pol. when assessing these target blood components and target analytes under optimized conditions.

**Figure 8:**
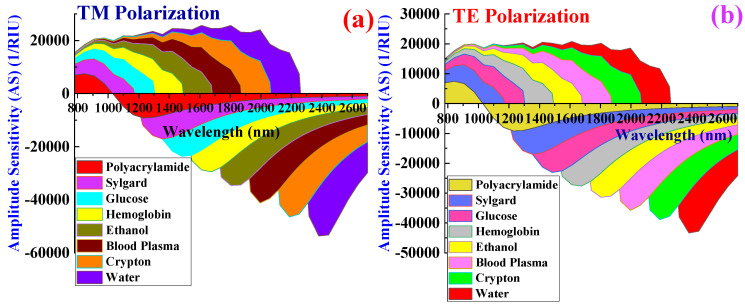
Variation in amplitude sensitivity for blood components and target analytes. (**a**) TM pol. (**b**) TE pol.

The amplitude interrogation method is used to calculate the *S_A_* of the biosensor. This approach can provide details about changes in light amplitude due to SPR effects, and it is calculated with (Equation (8)) [55].

(8) $$S_{A}\left({RIU}^{-1}\right)=-(1/\alpha_{CL})\times ∆\alpha_{CL}/∆RI$$

where ∆*α_CL_* represents the change in the *α_CL_*. Figure 8a,b shows the *S_A_* for TM pol. and TE pol.

The *S_A_* values for TM pol. corresponding to PA, sylgard, glucose, Hb, ethanol, plasma, crypton, and water are 15,428, 23,648, 36,722, 44,621, 52,684, 59,722, 63,488, and 71,224 RIU^−1^ respectively. Similarly, the *S_A_* values for TE pol. corresponding to PA, sylgard, glucose, Hb, ethanol, plasma, crypton, and water are 9848, 18,742, 26,422, 34,411, 38,622, 43,178, 51,224, and 58,112 RIU^−1^ respectively. The highest *S_A_* was obtained for water in both polarization modes. High *S_A_* values contribute to superior biosensor resolution.

Sensor resolution (*S_R_*) represents the ability of a biosensor to discern extremely minor variations in the RI of blood components. It can be determined using (Equation (9)) [55].

(9) $$S_{R}(RIU)=∆RI\times {∆\lambda }_{min}/∆\lambda_{p}$$

where ${∆\lambda }_{min}$ is the minimum spectral resolution of 0.1 nm. The calculated *S_R_* values for water, crypton, plasma, ethanol, Hb, glucose, sylgard, and PA for TM pol. are 5.84 × 10^−6^, 5.78 × 10^−6^, 5.29 × 10^−6^, 9.91 × 10^−6^, 9.47 × 10^−6^, 7.50 × 10^−6^, 8.46 × 10^−6^, and 6.10 × 10^−6^ RIU, respectively. Similarly, the *S_R_* values for water, crypton, plasma, ethanol, Hb, glucose, sylgard, and PA for TE pol. are 6.71 × 10^−6^, 6.57 × 10^−6^, 5.95 × 10^−6^, 1.12 × 10^−5^, 1.08 × 10^−5^, 1.51 × 10^−5^, 1.06 × 10^−5^, and 6.71 × 10^−6^ RIU, respectively. The relationship between the change in RI and the shift in RW is important from the perspective of biosensor optimization.

**Figure 9:**
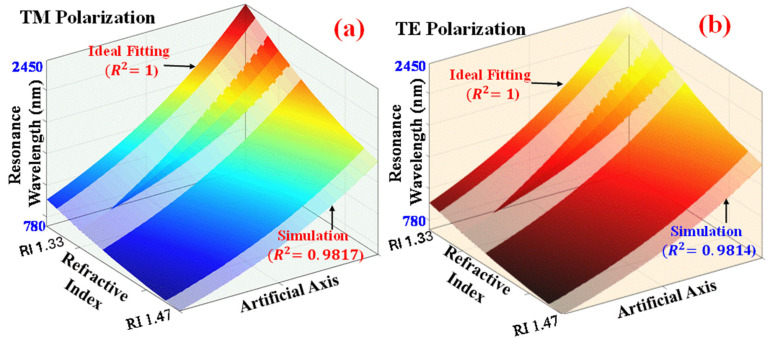
Polynomial fitting of RW with RI for various blood components. (**a**) TM pol. (**b**) TE pol.

Finally, the relationship between RW and RI for different blood components is assessed for both TM pol. and TE pol. as shown in Figure 9a,b, respectively. The coefficient of determination (R^2^), degrees of freedom for error (DFE), and root mean square error (RMSE) fitting parameter values are 0.9817, 2.68, and 0.0019, respectively, for TM pol., while those for TE pol. are 0.9814, 2.96, and 0.0017, respectively.

Therefore, for both TE pol. and TM pol., the R^2^; values are close to 1, highlighting a strong degree of correlation between the sensor parameters and the designed model.

### 3.2. Sensing Parameter Evaluation Through Increases in Plasmonic Material Thickness Beyond Optimal Levels 

**Figure 10:**
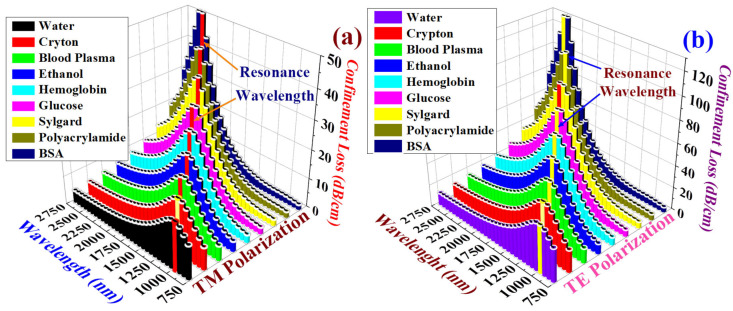
*α_CL_* behavior of the blood components and target analytes after increasing the thickness of plasmonic material. (**a**) TM pol. (**b**) TE pol.

Next, we explored how the sensing parameters *Sw* and *S_A_* vary in response to increasing the total width of the plasmonic materials beyond their optimal thickness. The combined thickness of the plasmonic materials Au and TiO_2_ rose from 45 nm and 85 nm to 50 nm and 90 nm, respectively. The *α_CL_* values corresponding to TM pol. and TE pol. with increased plasmonic material thickness when assessing blood components and target analytes are shown in Figure 10a,b. Table 3 provides an analysis of these graphs, detailing the observed variations in *α_CL_* and in RW for both polarization modes. The goal of these analyses is to clarify the effects of these changes on biosensor performance and operational efficiency.

**Figure 11:**
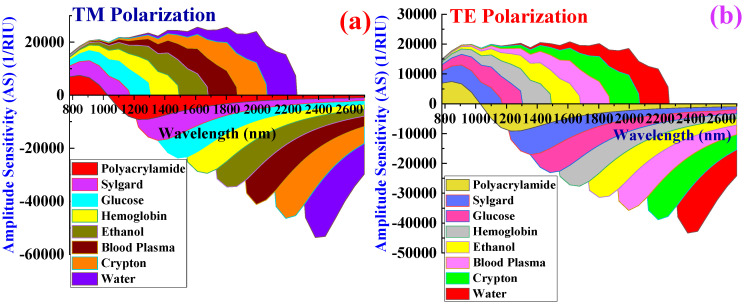
Change in amplitude sensitivity for various blood component and target analytes. (**a**) TM pol. (**b**) TE pol.

Figure 10a,b reflect the change in the *α_CL_*(dB/cm) for TM pol. and TE pol., respectively, under conditions of increased thickness, as presented in Table 3. The calculated *Sw* values for TM pol. corresponding to water, crypton, plasma, ethanol, Hb, glucose, sylgard, and PA are 15,200, 15,100, 16,800, 9450, 9900, 1266.66, 10,227.27, and 13,833.33 nm RIU^−1^, respectively. Similarly, the *Sw* values for TE pol. corresponding to water, crypton, plasma, ethanol, Hb, glucose, sylgard, and PA are 13,800, 14,800, 16,200, 8700, 9000, 6266.66, 9136.36, and 13,500 nm RIU^−1^, respectively. The average values of *Sw* for TM pol. and TE pol. are 11,472.15 nm RIU^−1^ and 11,425.37 nm RIU^−1^, respectively. Elevated *α_CL_* values can result in decreased *Sw*. This is due to the biosensor potentially exhibiting a diminished shift in RW in response to changes in the RI of blood components and target analytes. Such a decrease in *Sw* could complicate the detection and quantification of minor fluctuations in blood components. Consequently, *Sw* exhibits a significant decline when the plasmonic materials’ total thickness surpasses the ideal limit. *S_A_* values calculated when assessing the performance of this biosensor with increased plasmonic material thickness are shown in Figure 11a,b.

*S_A_* values of 9847, 18,422, 24,628, 29,142, 38,722, 46,842, 52,413, and 58,911 RIU^−1^ are measured for PA, sylgard, glucose, Hb, ethanol, plasma, crypton, and water, respectively, for TM pol. Corresponding *S_A_* values of 8462, 18,422, 21,243, 29,433, 35,418, 38,122, 40,034, and 45,844 RIU^−1^ are obtained corresponding to PA, sylgard, glucose, Hb, ethanol, plasma, crypton, and water, respectively, for TE pol.

The highest *S_A_* is achieved for water for both polarizations, but this peak *S_A_* was considerably lower than that achieved when using plasmonic materials of optimal thickness. The observed decrease in *S_A_* is attributable to an increase in *α_CL_*, which in turn leads to a reduction in *S_A_*. 

*S_A_* reflects the degree to which the intensity of the reflected or transmitted light is altered in response to RI changes in blood components. With a high *α_CL_*, a smaller amount of light is retained within the core, resulting in diminished amplitude changes in terms of the biosensor’s output for a given RI variation. Lastly, the sensing parameter *S_R_* was also expected to decline. Since we know that *S_R_* and *S_W_* can be related with each other as expressed by Equation (11).

(10) $$S_{R}\left(RIU\right)={∆\lambda }_{min}\times ∆RI/∆\lambda_{p}={∆\lambda }_{min}/S_{w}$$

(11) $$S_{R}\left(RIU\right)\propto 1/S_{w}$$

*S_R_* is inversely proportional to *S_W_*, when the plasmonic thickness exceeds its optimum value, the phase-matching between the core-guided mode and the SPP mode weakens, resulting in reduced evanescent field interaction and a lower *S_W_*. Consequently, *S_R_* increases (i.e., becomes worse), meaning the sensor can no longer detect the same minimal RI change as before. Similarly, excessive thickness can broaden the resonance curve and lower *S_A_*, further reducing the accuracy of RI detection. Therefore, maintaining the plasmonic coating close to its optimal thickness is critical not only for maximizing sensitivity but also for achieving the best possible *S_R_.*

In summary, elevated *α_CL_* within a PCF-SPR sensor can detrimentally affect both *S_W_* and *S_A_*, leading to diminished ability to detect and quantify RI changes in blood components.

It is generally advantageous to minimize *α_CL_* to improve sensitivity and overall performance. The expected minimum *S_R_* on the order of 10^−6^ may decrease to 10^−5^. Similarly, other sensing parameters may also be adversely influenced by changes in the optimal geometrical configurations.

**Figure 12:**
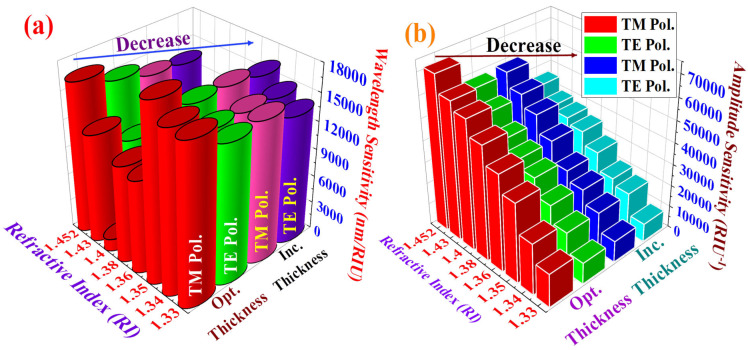
Comparison of sensing parameters at optimized and increased thickness levels: (**a**) wavelength sensitivity; (**b**) amplitude sensitivity.

Figure 12a,b offer a graphical comparison of the sensing parameters at optimized versus increased thickness of the plasmonic material. Augmenting the thickness of the plasmonic layer was found to result in a discernible drop in the values of these sensing parameters, marking a substantial decline in their characteristic measurements. By employing dual-mode analyses of both TM pol. and TE pol., this study successfully attained high *S_W_* and *S_A_* values from the kagome biosensor configuration, showcasing its proficiency as a means of detecting minute variations under ambient conditions. We measured *S_R_* values in the range of 10^−6^, demonstrating a high level of sensitivity. To evaluate the real-world applicability of our biosensor, we examined its ability to identify various blood components and target analytes. Table 4 presents a detailed comparison between the performance parameters for our biosensor and those used for blood component detection in prior studies.

## 4. Conclusions

In this study, a successfully designed, analyzed, and optimized a PCF-SPR biosensor, leveraging the kagome structure and SPR technology to efficiently detect a variety of blood components and target analytes is presented. The biosensor demonstrated proficiency in the identification of water, glucose, crypton, plasma, sylgard, ethanol, PA, Hb, and BSA based on shifts in RW. Utilizing a dual-polarization approach to evaluate both TM and TE modes, the *S_W_, S_A_* and *S_R_* for each mode were measured. The proposed sensor exhibits outstanding performance, achieving maximum *S_W_* of 18,900 nm RIU^−1^ for TM pol. and 16,800 nm RIU^−1^ for TE pol. corresponding to analyte plasma. Additionally, peak *S_A_* of 71,224 RIU^−1^ for TM pol. and 58,112 RIU^−1^ for TE pol. were recorded for analyte water. The highest *S_R_* for both polarizations is on the order of 10^−6^ RIU, highlighting its exceptional precision. The findings highlight the high sensitivity and advanced design of this biosensor, underscoring its significant potential as an effective tool for the analysis of blood components and associated target analytes. While the proposed kagome-lattice PCF SPR sensor demonstrates high sensitivity and robustness in simulation, several practical considerations must be acknowledged. Fabrication of complex kagome structures with precise elliptical air-hole geometries and optimal plasmonic coating thickness may incur relatively high costs, especially for small-scale production. Long-term stability can be influenced by environmental factors such as temperature fluctuations, humidity, and surface contamination of the plasmonic layer, potentially requiring protective coatings or periodic recalibration. Furthermore, batch-to-batch variability in the stack-and-draw fabrication process could lead to dimensional deviations that impact sensitivity and resolution. Addressing these limitations through cost-effective fabrication methods, protective surface treatments, and tighter manufacturing tolerances will be a priority in future work, alongside the planned miniaturization of the sensor. Future studies will focus on miniaturizing this biosensor for portable applications, enhancing its sensitivity, and broadening the range of detectable blood components and associated analytes to enable more comprehensive diagnostics.

## Figures and Tables

**Table 1:** Refractive indices of blood components and target analytes.

Blood Biomarker and Target Analytes	Refractive Index	Ref.
Water	1.330	[64]
Crypton	1.340	[65]
Plasma	1.350	[66]
Ethanol	1.360	[67]
Hemoglobin (Hb)	1.380	[68]
Glucose (40 gm/100 mL)	1.400	[1]
Sylgard	1.430	[65]
Polyacrylamide (PA)	1.452	[1]
Bovine Serum Albumin (BSA)	1.470	[1]

**Table 2:** Primary outcomes for TM pol. and TE pol.

Blood Components and Target Analytes	TMpol.		TEpol.		Shift	
	*α_CL_*	RW	*α_CL_*	RW	Δ*α_CL_*	|RW|
Water	3.48	841	8.74	862	5.26	21
Crypton	4.39	1012	10.47	1011	6.08	01
Plasma	6.43	1185	15.63	1163	9.20	22
Ethanol	7.48	1374	18.42	1331	10.94	43
Hb	8.64	1576	26.42	1509	17.78	67
Glucose	9.52	1787	32.42	1693	22.90	94
Sylgard	14.32	1827	48.96	1891	34.64	64
PA	19.42	2087	63.82	2097	44.40	10
BSA	24.82	2382	79.84	2365	55.02	17

**Table 3:** Primary outcomes TM pol. and TE pol.

Blood Components	TM pol.		TE pol.		Shift	
	*α_CL_*	RW	*α_CL_*	RW	*α_CL_*	|RW|
Water	07.12	982.0	18.11	991.0	10.99	09
Crypton	08.52	1134	24.12	1129	15.60	05
Plasma	11.63	1285	34.42	1277	22.79	08
Ethanol	16.47	1453	44.34	1439	27.87	14
Hb	22.18	1642	56.18	1613	34.00	29
Glucose	29.16	1842	75.11	1987	45.95	145
Sylgard	34.45	1878	89.18	2175	54.73	297
PA	39.42	2103	99.52	2376	60.10	273
BSA	48.12	2352	123.4	2619	75.28	267

**Table 4:** Comparison of sensing parameters with previously reported biosensors.

Modes/Year Design/Ref.	Blood Components	*S_W_* (nm RIU^−1^)	*S_A_* (RIU^−1^)	*S_R_* (RIU)
TM pol./ 2022/ EMD/ [29]	Water- Plasma	2000	249.1	5.0 × 10^−5^
	Plasma- WBC	3000	333.2	3.3 × 10^−5^
	WBC-Hb	4400	574.3	2.2 × 10^−5^
	Hb-RBC	12,400	NA	8.6 × 10^−6^
TM pol. TE pol./ 2022/ EMD/ [69]	Water	1750	2576.38	5.7 × 10^−5^
	Plasma			
	WBC			
	Hb			
	RBC			
	Water	1950	5078.99	5.1 × 10^−5^
	Plasma			
	WBC			
	Hb			
	RBC			
TM pol. 2022/ IMD/ [70]	RBC	6680	5663	NA
	WBC			
	Hb			
	Plasma			
	Water			
TE pol./ 2022/ IMD/ [70]	RBC	6930	5623	NA
	WBC			
	Hb			
	Plasma			
	Water			
Proposed Work TM pol. 2025/ EMD Design Kagome Model	Water	17,100	71,224	5.8 × 10^−6^
	Crypton	17,300	63,488	5.7 × 10^−6^
	Plasma	18,900	59,722	5.2 × 10^−6^
	Ethanol	10,100	52,684	9.9 × 10^−6^
	Hb	10,550	44,621	9.4 × 10^−6^
	Glucose	1333.33	36,722	7.5 × 10^−6^
	Sylgard	11,818.18	23,648	8.4 × 10^−6^
	PA	16,388.88	15,428	6.1 × 10^−6^
Proposed Work TE pol. 2025/ EMD Design Kagome Model	Water	14,900	58,112	6.7 × 10^−6^
	Crypton	15,200	51,224	6.5 × 10^−6^
	Plasma	16,800	43,178	5.9 × 10^−6^
	Ethanol	8900	38,622	1.1 × 10^−5^
	Hemoglobin	9200	34,411	1.0 × 10^−5^
	Glucose	6600	26,422	1.5 × 10^−5^
	Sylgard	9363.63	18,742	1.0 × 10^−5^
	PA	14,888.88	9848	6.7 × 10^−6^

## Data Availability

Datasets generated during the current study are available from the corresponding author on reasonable request.

## Supplementary Materials

The following supporting information can be downloaded at: https://www.mdpi.com/article/10.3390/bios15080539/s1, Table S1: Optimum dimension of the semi-major axis and semi-minor axis; Table S2: Variation in the confinement loss of the proposed sensor; Table S3: Variation in the wavelength sensitivity of the proposed sensor; Table S4: Variation in the amplitude sensitivity of the proposed sensor; Video S1: Electromagnetic field distributions of various TM and TE modes.
