# Supplementary material for: Numerical Study of a Novel Kagome-Inspired Photonic Crystal Fiber-Based Surface Plasmon Resonance Biosensor for Detection of Blood Components and Analytical Targets
Source: Biosensors (Basel). 2025 Aug 15;15(8):539. doi: 10.3390/bios15080539 (PMC12384928; doi:10.3390/bios15080539)
Supplement: Supplementary file 1 [file biosensors-15-00539-s001.zip › biosensors-3769188-supplementary-proofreading.pdf]

Supplementary Material

Numerical Study of a Novel Kagome-Inspired Photonic Crystal Fiber-Based Surface Plasmon Resonance Biosensor for Detection of Blood Components and Analytical Targets

Ayushman Ramola[1], Amit Kumar Shakya[1*], Ali Droby[2], Arik Bergman[1]

[1] Dept. of Electrical and Electronics Engineering, Ariel University, Ariel, Pin-40700, Israel (ayushmanr@ariel.ac.il; arikb@ariel.ac.il)
[2] Physics Program, Graduate Center of the City University of New York, New York (NY), Pin-10016, United States (adroby@gradcenter.cuny.edu)
* Correspondence: xlamitshakya.gate2014@ieee.org

Effect on sensing parameters by varying the optimum dimensions of semi-major and semi-minor axis of elliptical air holes

The optimized semi-minor axes of the elliptical air holes are a_1=13.75 μm and a_2=13.90 μm, while the semi-major axes are b_1=18.75 μm and b_2=18.95 μm, respectively. The semi-major and semi-minor axes were then varied by ±5%, and the resulting effects on sensing parameters such as confinement loss, wavelength sensitivity, and amplitude sensitivity are presented in this supplementary work. This variation is analyzed for the analyte water having refractive index (RI) of 1.33 RIU corresponding to both TM pol. and TE pol., respectively.

Table S1. Optimum dimension of the semi-major axis and semi-minor axis.

**Semi major axis**	**Optimized**	**+5% variation**	**-5% variation**
b1	18.75 μm	19.6875 μm	17.8125 μm
b2	18.95 μm	19.8975 μm	18.0025 μm
**Semi minor axis**			
a1	13.75 μm	14.4375 μm	13.0625 μm
a2	13.90 μm	14.595 μm	13.205 μm

Table S2. Variation in the confinement loss of the proposed sensor.

**Variation in dimension**	**TM pol.**	**TE pol.**
-5%	3.53 dB/cm	8.87 dB/cm
Optimum	3.48 dB/cm	8.74 dB/cm
**+5%**	3.41 dB/cm	8.63 dB/cm

Table S3. Variation in the wavelength sensitivity of the proposed sensor.

**Variation in dimension**	**TM pol.**	**TE pol.**
-5%	17105 nm RIU^{-1}	14908 nm RIU^{-1}
Optimum	17100 nm RIU^{-1}	14900 nm RIU^{-1}
**+5%**	17083 nm RIU^{-1}	14879 nm RIU^{-1}

Table S4. Variation in the amplitude sensitivity of the proposed sensor.

**Variation in dimension**	**TM pol.**	**TE pol.**
-5%	71243 RIU^{-1}	58179 RIU^{-1}
Optimum	71224 RIU^{-1}	58122 RIU^{-1}
**+5%**	71186 RIU^{-1}	58074 RIU^{-1}

Analysis

The impact of these dimensional variations on the sensor's key performance parameters was analyzed for an analyte RI of 1.33 RIU under both TM and TE pol. are summarized as follows.

Confinement Loss: Slightly decreases when dimensions increase by 5%, with values changing from 3.53 to 3.41 dB/cm (TM pol.) and from 8.87 to 8.63 dB/cm (TE pol.). Conversely, a 5% decrease in dimensions results in a small increase in confinement loss.

Wavelength Sensitivity: Remains almost unchanged, with a slight decrease when dimensions increase by 5% (e.g., TM: 17,105 to 17,083 nm/RIU; TE: 14,908 to 14,879 nm/RIU), and a slight increase when dimensions decrease by 5%.

Amplitude Sensitivity: Shows a similar trend, with minor variations across ±5% dimensional changes. For TM pol., it varies from 71,186 to 71,243 RIU^{-1}, and for TE from 58,074 to 58,179 RIU^{-1}.

Overall, these results indicate that the sensor performance is relatively stable against ±5% fabrication tolerances in elliptical hole dimensions, demonstrating robustness in confinement loss, wavelength sensitivity, and amplitude sensitivity for both TM and TE pol. Similarly, assessment of different RI values can also be performed for the proposed sensor model and different dimension variations.
