# Supplementary figures and images for: Numerical Study of a Novel Kagome-Inspired Photonic Crystal Fiber-Based Surface Plasmon Resonance Biosensor for Detection of Blood Components and Analytical Targets
Source: Biosensors (Basel). 2025 Aug 15;15(8):539. doi: 10.3390/bios15080539 (PMC12384928; doi:10.3390/bios15080539)

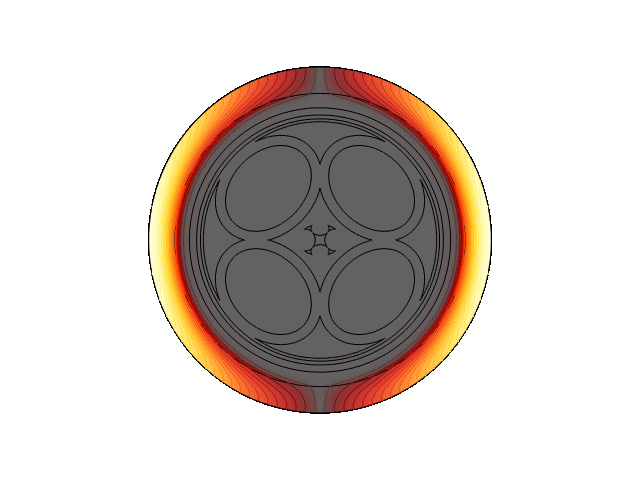

Supplement: Supplementary file 1 [file biosensors-15-00539-s001.zip › biosensors-3769188-Video S1.gif]
